# Analysis of Large Phenotypic Variability of EEC and SHFM4 Syndromes Caused by K193E Mutation of the TP63 Gene

**DOI:** 10.1371/journal.pone.0035337

**Published:** 2012-05-04

**Authors:** Jianhua Wei, Yang Xue, Lian Wu, Jie Ma, Xiuli Yi, Junrui Zhang, Bin Lu, Chunying Li, Dashuang Shi, Songtao Shi, Xinghua Feng, Tao Cai

**Affiliations:** 1 Department of Oral and Maxillofacial Surgery, School of Stomatology, the Fourth Military Medical University, Xi’an, Shaanxi Province, People's Republic of China; 2 Department of Oral Biology, School of Stomatology, the Fourth Military Medical University, Xi’an, Shaanxi Province, People's Republic of China; 3 Department of Paediatric Dentistry, School of Stomatology, the Fourth Military Medical University, Xi’an, Shaanxi Province, People's Republic of China; 4 Department of Dermatology, Xijing Hospital, the Fourth Military Medical University, Xi’an, Shaanxi Province, People's Republic of China; 5 Center for Genetic Medicine Research and Department of Integrative Systems Biology, Children’s National Medical Center, The George Washington University, Washington, D.C., United States of America; 6 Center for Craniofacial Molecular Biology, University of Southern California School of Dentistry, Los Angeles, California, United States of America; 7 Oral Infection and Immunity Branch, National Institute of Dental and Craniofacial Research, National Institutes of Health, Bethesda, Maryland, United States of America; University of Tampere, Finland

## Abstract

EEC (ectrodactyly, ectodermal dysplasia, clefting; OMIM 604292) is an autosomal dominant developmental disorder resulting mainly from pathogenic mutations of the DNA-binding domain (DBD) of the TP63 gene. In this study, we showed that K193E mutation in nine affected individuals of a four-generation kindred with a large degree of phenotypic variability causes four different syndromes or TP63-related disorders: EEC, Ectrodactyly-ectodermal dysplasia (EE), isolated ectodermal dysplasia, and isolated Split Hand/Foot Malformation type 4 (SHFM4). Genotype-phenotype and DBD structural modeling analysis showed that the K193-located loop L2-A is associated with R280 through hydrogen bonding interactions, while R280 mutations also often cause large phenotypic variability of EEC and SHFM4. Thus, we speculate that K193 and several other DBD mutation-associated syndromes may share similar pathogenic mechanisms, particularly in the case of the same mutation with different phenotypes. Our study and others also suggest that the phenotypic variability of EEC is attributed, at least partially, to genetic and/or epigenetic modifiers.

## Introduction

The TP63 gene encodes the tumor suppressor p63 with six isoforms that are involved in limb, epithelial, and craniofacial development. Heterozygous mutations of the TP63 gene have been found to be responsible for at least five rare syndromic and two non-syndromic human disorders [1]–[6]. The full-length p63 consists of six domains: 1) the transactivation (TA) domain, 2) the DNA binding domain (DBD), 3) the tetramerization (ISO) domain, 4) the second transactivation domain (TA2) domain, 5) the sterile-α-motif (SAM), and 6) the transactivation inhibitory domain (TID). The DBD is present in all splicing isoforms of p63, but its mutations result in multiple syndromes, such as the most prevalent ectrodactyly-ectodermal dysplasia-clefting syndrome (EEC), the ectrodactyly-ectodermal dysplasia syndrome (EE), the isolated Ectodermal dysplasia, and the isolated split hand/foot malformation type 4 (SHFM4) [2], [7]. The main difference between EEC and SHFM4 is that three major symptoms (split-hand/foot, ectodermal dysplasia, and cleft lip/palate) are observed in EEC patients [8], whereas only limb malformations (split-hand/foot) are found in SHFM4 patients [9], [10]. However, whether the genotype–phenotype correlation of these two disorders is associated with the structure and function of DBD remains unclear.

In recent crystal structure analysis of p63 DBD, of 22 p63 DBD mutated positions, four are expected to impair DNA binding, 16 are expected to reduce protein stability, while others are thought to indirectly impact DNA binding, zinc binding, or tetramerization [11]. However, it is still unclear whether the different phenotypes between EEC and SHFM4 are associated with the p63 DBD mutations in different positions or are simply due to different genetic modifiers that result in wide phenotypic variability.

In this study, we describe a wide phenotypic spectrum featured in EEC, EE, isolated Ectodermal dysplasia, and also SHFM4 observed from a Chinese family. Phenotypic variations and structural models of p63 DBD with several specific mutations are also discussed.

## Results

### Phenotypic Variability in the Affected Family Members

**Figure 1:**
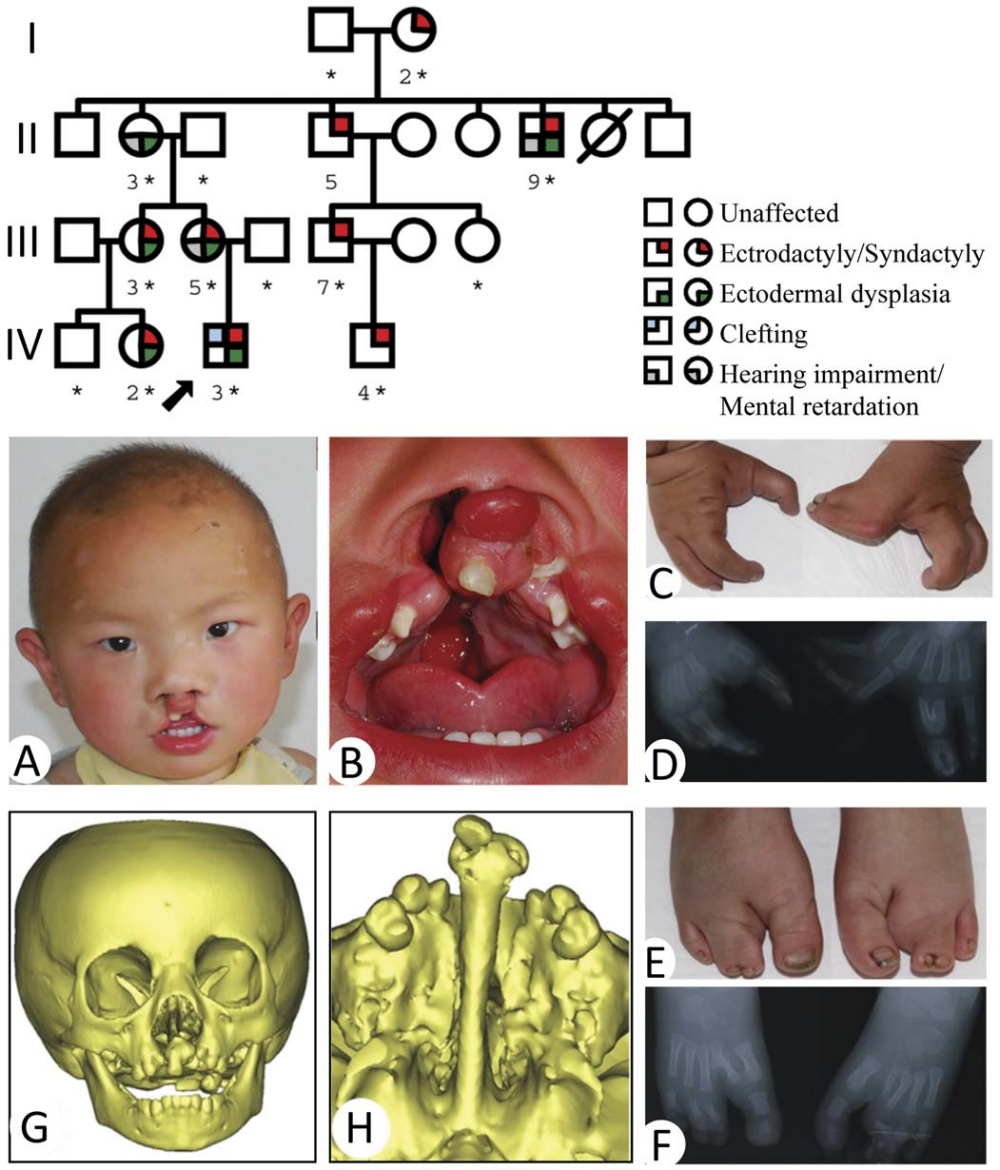
The pedigree of the family and clinical feature of the proband. Upper panel: Filled symbols represent the affected individuals. The proband is indicated by an arrow. Males are represented by squares, females by circles. A slashed circle indicates a deceased subject. The person whose DNA sample is available for sequencing is marked by “*”. For simplicity, spouses and children of normal siblings in generation II and III are not shown. Lower panel: Clinical feature of the proband. (A) Thinning of hair on the scalp and lateral eyebrows, ocular hypertelorism, and cleft lip. (B) Cleft palate. (C) Syndactyly and ectrodactyly of the hands. (D) Radiography of the hands. (E) Malformation of the feet. (F) Radiography of the feet. (G & H) CT scanning and reconstruction of the head and craniofacial bones.

**Figure 2:**
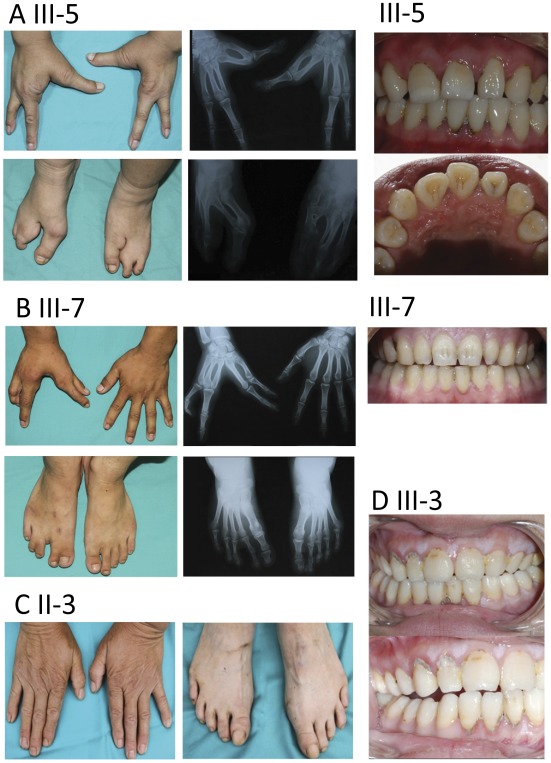
Three types of hands/feet manifestations and malformed teeth. (A) Symmetrical deformities. Patient III-5: absence of 2nd–3rd fingers and 2nd–3rd toes. Hand radiography: absence of 2nd–3rd phalanges and fusions of the 1st–2nd and 3rd–4th metacarpale in both hands. Feet radiography: absence of 2nd–3rd phalanx and osseous fusion of 1st–2nd metatarsal bone. Left panel: spade-shaped central incisor and open bite. (B) Asymmetric deformities. Patient III-7: Syndactyly of 1st–2nd finger, absence of middle finger, syndactyly of 1st–2nd and 3rd–4th toes. Radiography: missing third phalanges and cutaneous syndactyly of 1st–2nd and 3rd–4th toes. Left panel: cone-shaped left maxillary lateral incisor. (C) Patient II-3: Hands or feet were undistinguished from normal controls. (D) Patient III-3: Absent right maxillary lateral incisor and malformed lingual fossa in the left maxillary lateral incisor.

Clinical analysis revealed that ectrodactyly and syndactyly in hands and/or feet were present in almost all affected individuals with the exception of patient II-3 who showed an isolated ectodermal dysplasia. Three types of hands/feet malformation were found in these patients: 1) Symmetrical Split hand-Split foot malformation (shown in patient IV-3, Figure 1; patient III-5, Figure 2A; patient II-9, Figure S1A; patient III-3, Figure S1B; and patient IV-4, Figure S1C). 2) Asymmetric Split hand-Split foot (shown in patient III-7, Figure 2B). 3) Minor abnormalities (or none at all) in hands or feet (shown in patient II-3, Figure 2C; patient I-2, Figure S1D; and patient IV-2, Figure S1E). In addition, patient IV-2 showed syndactyly on her right foot, but had totally normal hands and left foot. Interestingly, patient II-3 appeared to have hearing impairment and mild mental retardation; but no deformities were found in her hands or feet (Figure 2C). Both of her daughters (III-3 and III-5) had symmetrical hands/feet abnormalities.

Overall, the main deformities related to the ectodermal development in this family includes split hands/feet (8/9) and several other ectodermal developmental dysplasia, such as hypopigmented and sparse hair (6/8), sparse eyebrows (6/8), and ocular hypertelorism (2/8) (Table S2). Central nervous system (CNS) defects such as mild mental retardation (3/7) and hearing impairment with or without speech impairment (2/8) were also observed (Table S2). Interestingly, the CNS defects were the main clinical manifestation for patient II-3, while she did not show any limb defect. No family members showed any significant abnormalities in skin, eyes, mouth, nose, nipples, or exocrine glands (for sweat), lacrimal, and salivary secretion. Nail deformity was shown in the patient II-9, but not present in other family members. Table S2 also listed several alterations related to oral-craniofacial development, including micrognathia (1/9), bilateral cleft lip-palate (1/9), malformed teeth (3/8), and hypodontia (4/8) (Figure 2). Taken together, typical EEC phenotypes were observed in patient IV-3, EE phenotypes in II-9, III-3, III-5, and IV-2, possible isolated SHFM4 phenotypes in I-2, III-7, and IV-4, and isolated Ectodermal dysplasia in II-3 (Table S2).

### Mutation Analysis

**Figure 3:**
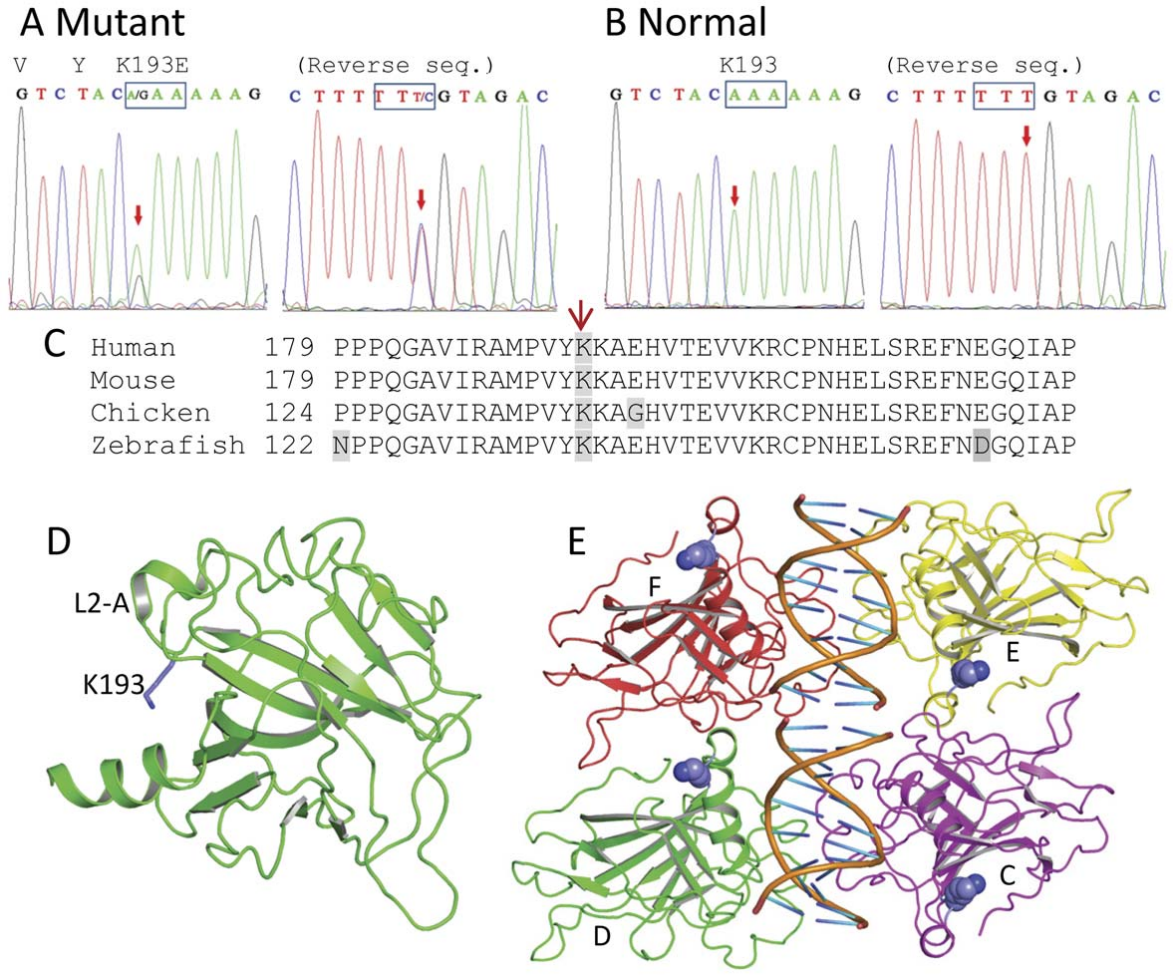
TP63 mutation and DBD structure. (A) A missense mutation K193E of p63 DBD in the proband with heterozygous A/G at nucleotide 577. (B) Sequence of exon 5 in normal control. (C) Multiple sequence alignment. K193 of human p63, indicated by the arrow, is evolutionarily conserved as shown in four representative species. Start position of each amino acid sequence is labeled. (D) Location of K193 (shown in blue stick) is located on the protein surface and at the loop L2-A in the crystal structure of p63 DBD monomer (shown in green ribbons, subunit D of PDB 3QYM). (E*)* K193 (shown in blue space-filling models) is located in the dimer-dimer interface of the type II tetramer (subunits C, D, E, and F of PDB 3QYM). Different subunits are shown as ribbon diagrams in different colors. Mutation of K193 will potentially affect the formation of the type II tetramer.

PCR products of the TP63 gene were bidirectionally sequenced from two affected individuals (III-5 and IV-3). A heterozygous missense mutation 577A→G in exon 5 was detected, which predicts amino acid substitution p. K193E (TA p63 alpha isoform, GenBank acc. no., AF075430) (Figures 3A and 3B). Further analysis of this exon for the family confirmed the K193E mutation present in all patients examined, but not in unaffected individuals. In order to exclude the possibility of a rare single nucleotide polymorphism (SNP) in the Han population, 50 normal unrelated individuals were sequenced and no mutations were found. Multiple sequence alignment showed that K193 in the p63 DBD is evolutionarily conserved from zebrafish to humans (Figure 3C).

### Location of Mutated Residues in 3D Structure

Crystal structural analysis of p63 DBD suggests that most EEC mutations that cause developmental defects either reduce the protein stability or affect DNA binding ability [11]. In the monomer of p63 DBD, K193 is on the loop L2-A (Figure 3D); while when the DBD is in the complex with target elements of DNA, K193 is on the outer surface and the dimer-dimer interface of type II tetramer (Figure 3E). K193 mutation may thus affect p63 association with other proteins and the dimer-dimer stability, but not the DNA binding [11].

Since K193E mutation was previously identified in a male fetus whose father showed isolated split-hand/foot (SHFM4) phenotypes [12] and was claimed as a SHFM4-specific mutation, we thus position all other SHFM4-associated residues in 3D structures for a potential correlation analysis. In Figure S2A, K194 is expected to be located next to K193 on the loop L2-A; R280 is also associated with the L2-A loop, unexpectedly, by hydrogen bonding interaction with E200, while K193 is associated with S10 region via hydrogen bonding interaction with E302. These interactions suggest that the mutations (K193E, K194E, and R280H/C/S) might affect protein-protein interactions via the L2-A loop-containing region. However, positions of T154–Y155 and G310 are found to be associated with DNA-binding. Therefore, these two mutations (proline insertion between residue T154–Y155 and G310E) are expected to affect DNA affinity directly (Figure S2B). Whether these mutations affect protein interactions or DNA affinity needs further investigation.

## Discussion

Review of the previously reported EEC patients [13]–[17] and SHFM4 patients with six different DBD mutations [2], [13], [18], including K193E [19], K194E [7], [19], [20], R280C, R280H (but not R280/S that causes EEC) [7], [19], [21], [22], G310E [23], the proline insertion between T154–Y155 [19], and patients in this study, revealed a wide phenotypic spectrum of the TP63 gene mutations. The fact that different patients of this family, who carried the same mutation, showed a substantial degree of clinical variability indicates that K193E is a common EEC mutation, rather than a SHFM4-specific one as previously claimed.

It was observed that K193 was a potential SUMO-1 target [24] and K193E was less sensitive to Itch (E3-ubiquitin ligase)-mediated p63 degradation [25]. However, a recent study demonstrated that Itch directly binds the PY motif of p63 SAM domain rather than the DBD domain [26]. Since K193 is on the surface of DBD, it may interact with other proteins and is involved in the stability of the p63 tetramer. In addition, the mutant K193E may have dominant-negative effects and thus compromise the function of the wild-type allele of p63. How the expression level and stability of p63 are affected by K193E in our patients needs to be further studied to be confirmed.

During the course of our investigation, we found that four SHFM4 mutations (K193E; K194E, R280C, and R280H) are associated with the L2-A loop of p63 BDB and two SHFM4 mutations (proline insertion and G310E) are affecting DNA binding. However, six EEC mutations (Y192C/D, V202M, and R204L/Q/W) are also found on the L2-A loop (residue 192 to 205) and additional nine EEC mutations (S272N, R279C/H/Q, R304P/Q/W, and C308S/Y) are expected to impair DNA binding [9], [11]. Moreover, even the same residue mutations (K193E and R280C/H/S) are found to cause either EEC or SHFM or both. We speculate that global DBD structure and stability are expected to be affected by mutations of different residues that are tightly connected by side chains such as the L2-A loop. Therefore, the associated structural locations and functions, as well as the overlapping phenotypic spectrum, indicate that EEC and SHFM4 might be caused by similar pathogenic mechanisms. Further experimental p63 structure analyses via mutagenesis and functional tests are required to testify this hypothesis.

Although the causes of the wide intrafamilial and interfamilial phenotypic variability remain unclear, we agree that one of them could be the result of modifier genes and/or genetic background effect [27], particularly in the case of the same mutation with different phenotypes [e.g., R280C/H found in either EEC [21] or SHFM4 [7], R280S only found in EEC [19]]. What determines the different phenotypic expressions (EEC vs. SHFM) of the R280C/H/S or K193E mutation in different families or even in the same family? The influence of genetic and/or environmental modifiers has been suggested to explain the variable clinical expressivity [27]. Linkage analysis of an EEC family with R280C has identified at least two modifier genes for clefting phenotype on chromosomes 4q and 14 [21]. The Orofacial Cleft 4, which was mapped to the same region on 4q21–31 [28], could be one of the candidates. Also, loss of the p63 binding site, which is required for the expression of the distal-less homeobox 6 gene (DLX6) located in the SHFM1 locus on chromosome 7q, was recently identified in a patient with SHFM phenotype [29]. Therefore, the different clinical conditions exhibited in EEC/SHFM4 patients suggest that additional minor modifying genes that predispose to non-syndromic cleft lip/palate and other tissues/organs could presumably contribute to the expressivity of the equivalent phenotypes in different patients. On the other hand, hundreds of targets of p63 were identified by ChIP on chip analysis in human keratinocytes, including many critical transcriptional factors, such as Notch, TGFβ and WNT signaling in developmental pathways [30], [31]. As expected, a homozygous mutation of the WNT10b gene was recently identified as a new causative gene for SHFM [32]. These results suggest that genetic modifiers that participate or overlap the same signaling pathway in limb or craniofacial ectodermal development could compensate or reduce the phenotypes resulting from the dysfunctional p63. Further studies using SNP genome screening in large kindreds may identify modifying genes that influence variable expressivity of monogenic disorders.

## Materials and Methods

### Patients

Nine affected individuals from a four-generation family with autosomal dominant inherited patterns (located in Zhen’an county of Shaanxi province in Northwest China) were examined (Figure 1, upper panel). General information of the patients is provided in the Table S1. DNAs were extracted (QIAamp DNA blood kit, Qiagen, Chatsworth, CA, USA) from peripheral blood samples of available patients, normal members of this family, and 50 unrelated normal controls. The study was approved by the Ethics Committee, School of Stomatology of the Fourth Military Medical University. Written informed consents were obtained from each subject. Parents or guardians consented on the behalf of the patients who were under 18 year-old (IV-2, IV-3, and IV-4) or who had a reduced capacity/ability to consent (II-3, II-9, and III-5).

The proband is a 2.5-year-old boy who was presented with a bilateral cleft lip and palate, hypopigmented and sparse hair, and ocular hypertelorism (Figures 1A and 1B). He had syndactyly and ectrodactyly of both hands and feet. His right index and both middle fingers were absent while the 1st–2nd and 3th–4th fingers of both hands showed syndactyly (Figure 1C). Radiography showed aplasia in the 2nd metacarpale, 2nd–3rd phalanges to the right hand, and syndactyly on the 1st–2nd and 3rd–4th phalanges to his left hand (Figure 1D). Both of his 2nd toes were ectrodactyly, while the 3th–4th toes were syndactyly (Figure 1E). Radiographic analysis showed the absent of the 2nd phalanges, the cutaneous fusion to the 3rd–4th phalanges, as well as a transversal mesial phalanx of the 3rd ray in the right foot (Figure 1F). CT scanning and reconstruction of his craniofacial bones showed the hard palate and alveolar bone defects that are often seen in bilateral cleft lip and palate patients (Figures 1G, 1H).

### Mutation Screening

All exons and exon/intron splice junctions of the TP63 gene were PCR-amplified as described previously [33]. PCR products were purified with DNA Fragment Quick Purification Kit (DingGuo, Beijing, China) and sequenced (ABI 377 Sequencer, Perkin-Elmer, Norwalk, Connecticut, USA). Sequence variants were identified by MegAlign 5.01 (DNASTAR, Madison, USA) and confirmed by additional PCR samples and sequencing. Protein modeling was conducted based on the recent data of p63 DBD structure in Protein Data Bank (PDB ID codes 3QYM and 3QYN, http://www.pdb.org), and mutation-related residues were positioned in 3D structural models using PyMOL Molecular Graphics System, Version 1.3r1.

## Supporting Information

Figure S1 **Hands/feet manifestation in additional patients.** (A) Patient II-9 showed symmetrical deformities. His index and middle fingers and 2nd–3rd toes were absent. The finger nails were dystrophic. (B) Patient III-3 showed the absence of index and middle fingers, syndactyly of 1st–2nd and 3rd–4th toes of right foot, and the absence of 2nd toe and syndactyly of 3rd–4th toes in her left foot. (C) Patient IV-4 showed asymmetric split hands: missed middle finger of the right hand and syndactyly of the 3rd–4th fingers of left hand. But absence of 2nd–3rd toes was found in both feet. (D) Minor deformity. Patient I-2 only showed a small split in index finger of right hand and syndactyly of 2nd–4th toes in right foot. (E) Patient IV-2 only showed polydactyly or syndactyly of 2nd–3rd and 4th–5th toes in right foot. No deformity were found in both hands and left foot.
(TIF)

Figure S2 **Locations of mutated residues in DBD.** (A) Residue K193, K194 and R280 and their associations with L2-A loop of p63 DBD monomer. Side chains of residue K193, K194, and R280 are shown in blue sticks. Hydrogen bonding interactions of K193-E302 and R280-E200 were indicated by red dashed lines. Structure of p63 DBD is shown in green ribbons. L2-A loop is highlighted in red. (B) Residue G310 and T154, shown in light blue space-filling models, are close to each other in 3D structure and close to the bound DNA (shown in golden yellow stick model). The subunit C, D, E, and F are shown in different colored ribbons form type II tetramer in the crystal structure.
(TIF)

Table S1 **General information of nine patients in this study.**
(DOC)

Table S2 Head and oral-craniofacial phenotypes of nine patients.
(DOC)
