# Supplementary figures and images for: Analysis of Large Phenotypic Variability of EEC and SHFM4 Syndromes Caused by K193E Mutation of the TP63 Gene
Source: PLoS One. 2012 May 4;7(5):e35337. doi: 10.1371/journal.pone.0035337 (PMC3344828; doi:10.1371/journal.pone.0035337)

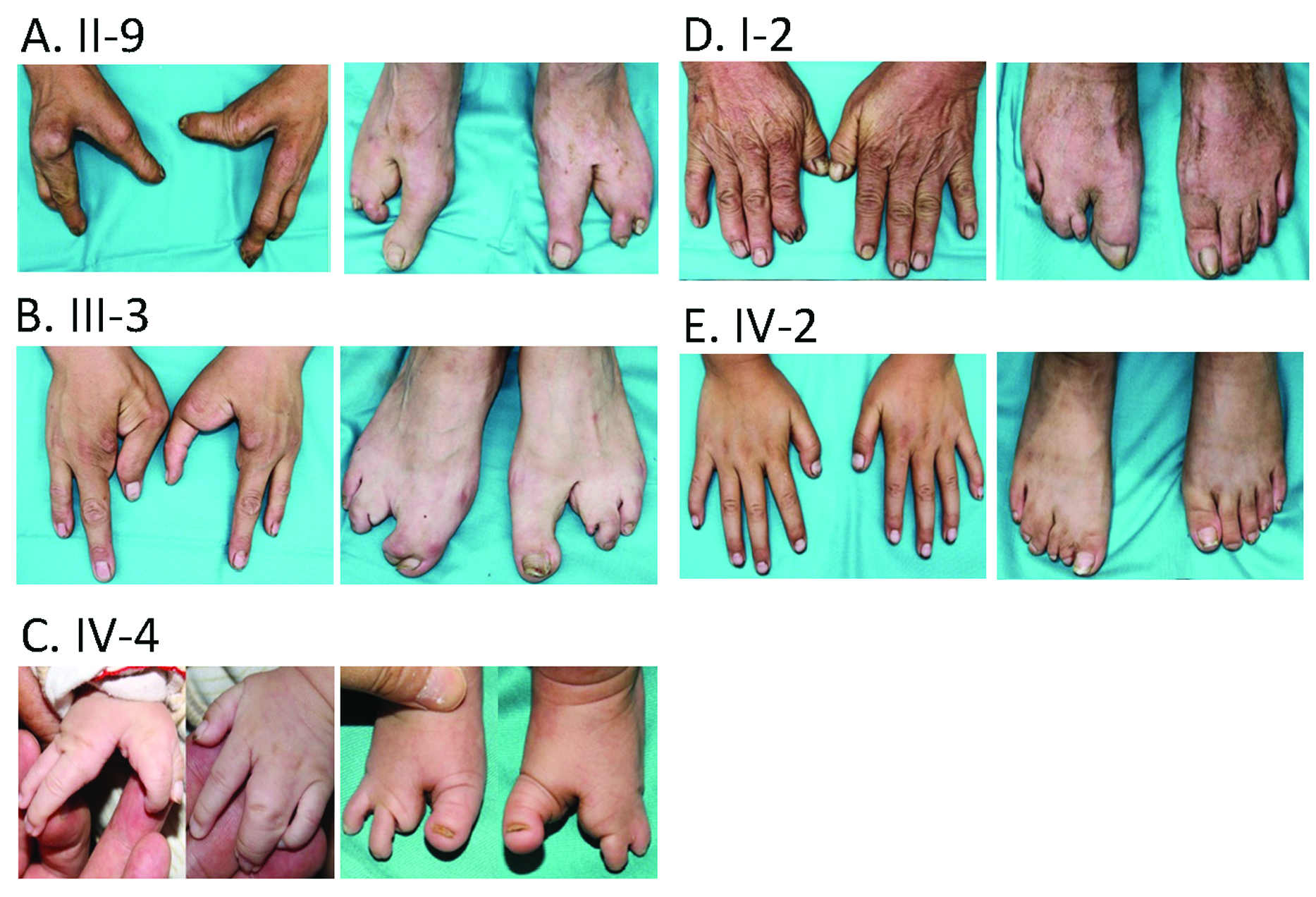

Supplement: Figure S1 — Hands/feet manifestation in additional patients. (A) Patient II-9 showed symmetrical deformities. His index and middle fingers and 2nd–3rd toes were absent. The finger nails were dystrophic. (B) Patient III-3 showed the absence of index and middle fingers, syndactyly of 1st–2nd and 3rd–4th toes of right foot, and the absence of 2nd toe and syndactyly of 3rd–4th toes in her left foot. (C) Patient IV-4 showed asymmetric split hands: missed middle finger of the right hand and syndactyly of the 3rd–4th fingers of left hand. But absence of 2nd–3rd toes was found in both feet. (D) Minor deformity. Patient I-2 only showed a small split in index finger of right hand and syndactyly of 2nd–4th toes in right foot. (E) Patient IV-2 only showed polydactyly or syndactyly of 2nd–3rd and 4th–5th toes in right foot. No deformity were found in both hands and left foot. (TIF) [file pone.0035337.s001.tif]

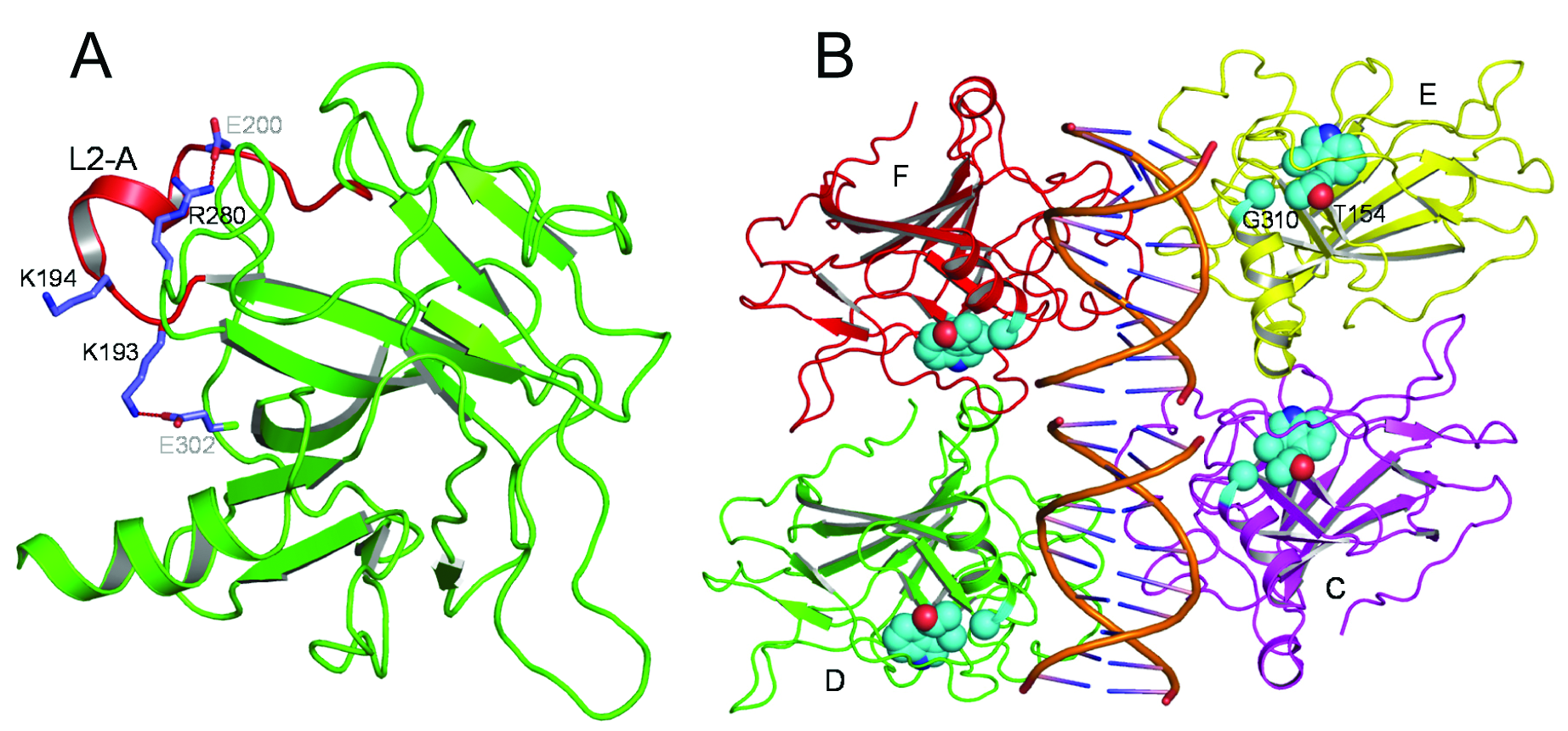

Supplement: Figure S2 — Locations of mutated residues in DBD. (A) Residue K193, K194 and R280 and their associations with L2-A loop of p63 DBD monomer. Side chains of residue K193, K194, and R280 are shown in blue sticks. Hydrogen bonding interactions of K193-E302 and R280-E200 were indicated by red dashed lines. Structure of p63 DBD is shown in green ribbons. L2-A loop is highlighted in red. (B) Residue G310 and T154, shown in light blue space-filling models, are close to each other in 3D structure and close to the bound DNA (shown in golden yellow stick model). The subunit C, D, E, and F are shown in different colored ribbons form type II tetramer in the crystal structure. (TIF) [file pone.0035337.s002.tif]
