# Supplementary material for: Analysis of Large Phenotypic Variability of EEC and SHFM4 Syndromes Caused by K193E Mutation of the TP63 Gene
Source: PLoS One. 2012 May 4;7(5):e35337. doi: 10.1371/journal.pone.0035337 (PMC3344828; doi:10.1371/journal.pone.0035337)
Supplement: Table S1 — General information of nine patients in this study. (DOC) [file pone.0035337.s003.doc]

**Table S1. General information of nine patients in this study**

| Patient code | I-2 | II-3 | II-9 | III-3 | III-5 | III-7 | IV-2 | IV-3 | IV-4 |
| --- | --- | --- | --- | --- | --- | --- | --- | --- | --- |
| Sex | F | F | M | F | F | M | F | M | M |
| Age (yr) | 67 | 43 | 37 | 23 | 21 | 21 | 4.5 | 2.5 | 0.1 |
| Birth/pregnancy | 7/8 | 2/2 | N/A | 2/2 | 1/2 | 1/1 | N/A | N/A | N/A |
| Height (cm) | 149 | 148 | 155 | 162 | 163 | 168 | 101 | 82 | N.D. |
| Weight (kg) | 52 | 49 | 44 | 50 | 50 | 60 | 17 | 11 | N.D. |
| Head circumference | 55 | 55 | 56 | 56 | 56 | 56 | 45 | 41 | N.D. |
| Medial canthus  distance (cm) | 3.4 | 3.5 | 3.8 | 3.7 | 4.3 | 4 | 3.9 | 3.8 | N.D. |
| Lateral canthus  distance (cm) | 10 | 10.5 | 10.5 | 11 | 10.8 | 11 | 10 | 9.5 | N.D. |

Note: N/A, Not applicable. N.D., not determined.
