# Supplementary material for: Analysis of Large Phenotypic Variability of EEC and SHFM4 Syndromes Caused by K193E Mutation of the TP63 Gene
Source: PLoS One. 2012 May 4;7(5):e35337. doi: 10.1371/journal.pone.0035337 (PMC3344828; doi:10.1371/journal.pone.0035337)
Supplement: Table S2 — Head and oral-craniofacial phenotypes of nine patients. (DOC) [file pone.0035337.s004.doc]

**Table S2. Head and oral-craniofacial phenotypes of nine patients**

| Patient code | I-2 | II-3 | II-9 | III-3 | III-5 | III-7 | IV-2 | IV-3 | IV-4 |
| --- | --- | --- | --- | --- | --- | --- | --- | --- | --- |
| Hypopigmented hair | - | Yes | Yes | Yes | Yes | - | Yes | Yes | N.D. |
| Ocular hypertelorism | - | - | - | - | - | - | Yes | Yes | N.D. |
| Sparse eyebrows | Yes* | Yes | - | Yes | Yes | - | Yes | Yes | N.D. |
| Micrognathia | - | Yes | - | - | - | - | - | - | - |
| Cleft lip | - | - | - | - | - | - | - | Yes | - |
| Cleft palate | - | - | - | - | - | - | - | Yes | - |
| Dentition | 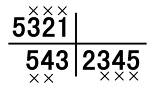 | 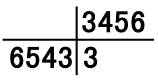 | 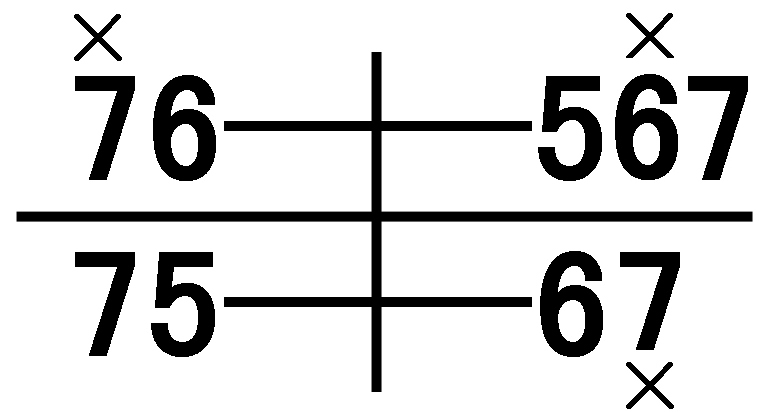 | 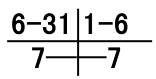 | 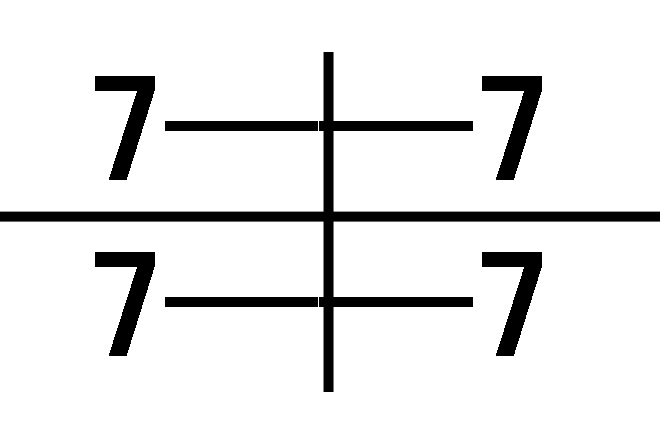 | 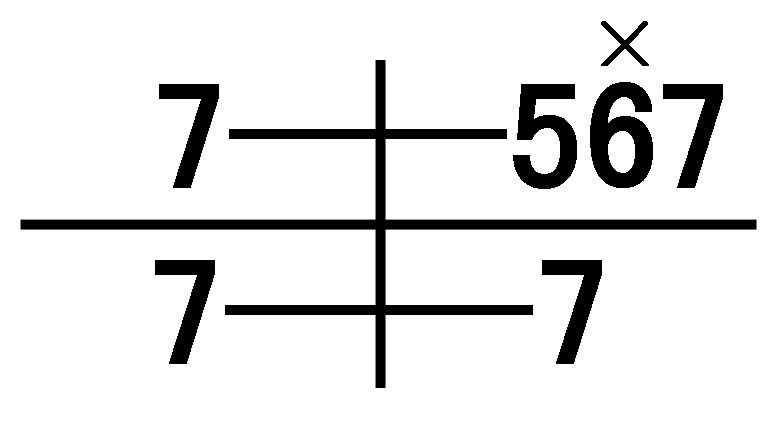 | 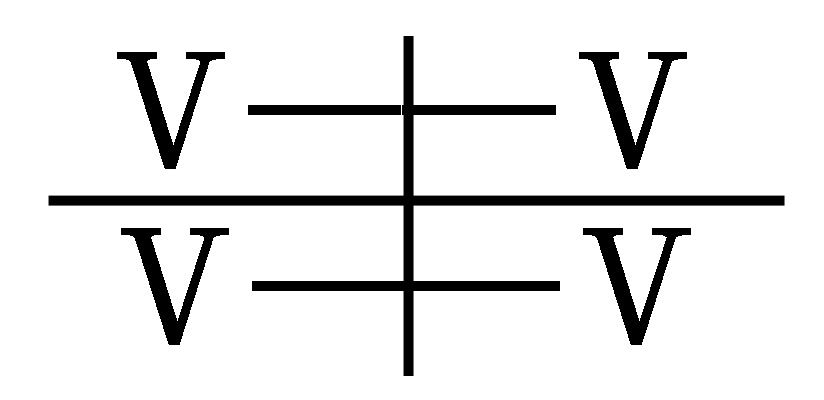 | 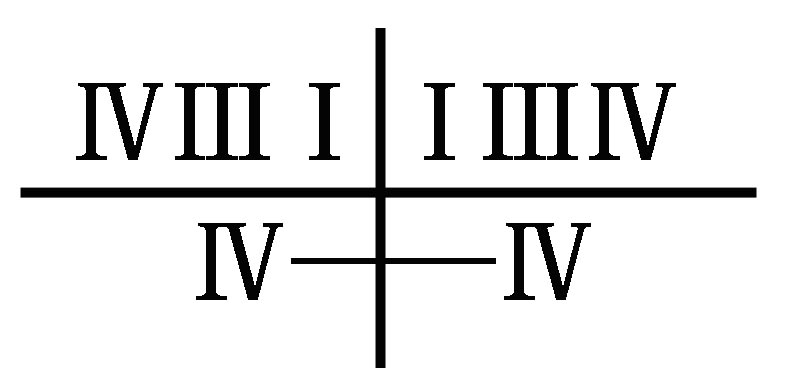 | N.D. |
| Hypodontia | - | - | C6 | A2 | - | - | - | E2 and F2 | N/A |
| Malformed teeth | - | - | - | Yes | Yes | Yes | - | - | N/A |
| Mental retardation | - | Yes | Yes | - | Yes | - | - | N.D. | N.D. |
| Hearing impairment | - | Yes | Yes/mute | - | - | - | - | - | N.D. |
| Clinical feature/diagnosis | SHFM4 | E | EE | EE | EE | SHFM4 | EE | EEC | SHFM4 |

Notes: -, normal. N/A, not applicable. N.D., not determined. II-5, not available for examination. Phenotypes such as low-set ears, ankyloblepharon, and dry mouth reported in other patients with EEC/SHFM were not found in this study. IV-4 is ~0.1 yr-old. Hearing impairment in II-3 started at 10-yr-old; hear impairment companying with speech impairment in II-9 at 6-yr-old. While none of the patients showed any skin damage, other phenotypes, such as hypopigmented hair, sparse eyebrows, hypodontia, and malformed teeth, may represent part of the ectodermal dysplasia in these patients. In addition, mental retardation (3/7 patients) were rarely seen in other patients with EEC, which may not be caused by the TP63 mutation. *, sparse eyebrows are not uncommonly seen in elderly persons. EEC, ectrodactyly-ectodermal dysplasia-clefting syndrome; SHFM4, isolated split hand/foot malformation type 4; E, isolated Ectodermal dysplasia; EE, ectrodactyly-ectodermal dysplasia syndrome.

Dentition: Permanent teeth Deciduous teeth

right top (A) left top (B) right top (E) left top (F)

right bottom (C) left bottom (D) right bottom (G) left bottom (H)

The cross lines divide the dentition of a person into four quadrants: right top, left top, right bottom, and left bottom; e.g. A, B, C, and D areas for permanent teeth; E, F, G, and H areas for deciduous teeth.

Arabic numerals represent permanent teeth: 1 means the central incisor, 7 means the second molar. Roman numerals represent the deciduous teeth:

I means the central incisor, V means the second molar.

The line (—) between numerals represents a contiguous dentition from one tooth to another. A missed number represents a lost tooth or hypodontia in that position. × means the residual root of the corresponding tooth.
